# Study on the kinetics of formation process of emulsion of heavy oil and its functional group components

**DOI:** 10.1038/s41598-024-59637-9

**Published:** 2024-04-18

**Authors:** Jinhe Liu, Chengdi Sun, Zengmin Lun, Yao Li, Xinyu Tang, Qingxuan Zhang, Pujiang Yang

**Affiliations:** 1 College of Chemistry and Chemical Engineering, China University of Petroleum, Qingdao, China; 2 Petroleum Engineering Technology Research Institute, Sinopec Shengli Oilfield Company, Dongying, China; 3 Experimental Research Center, Sinopec Petroleum Exploration and Production Research Institute, Beijing, China

**Keywords:** Conductivity, Heavy oil emulsion, The law of dynamic, Emulsification rate, Heavy oil active component, Reaction kinetics and dynamics, Crude oil

## Abstract

Enhanced oil recovery (EOR) by in situ formation of oil-in-water emulsion in heavy oil cold production technology has received growing interest from the petroleum industry. We present an experimental study of emulsification of model oils prepared by heavy oil and its functional group compositions dissolved into toluene brought into contact with a surfactant solution. The effects of functional group composition, emulsifier concentration, temperature, pH and stirring speed on the emulsification rate of heavy oil was investigated. A second-order kinetic model characterizing the temporal variation of conductivity during the emulsification has been established. The results show that acidic and amphoteric fractions exhibit higher interfacial activity, larger emulsification rate constant and faster emulsification rate. With the increase of emulsifier concentration, the emulsification rate constant increase to the maximum value at a concentration of 0.05 mol/L before decreasing. Temperature increase benefits the emulsification rate and the activation energy of the emulsification process is 40.28 kJ/mol. Higher pH and stirring speed indicate faster emulsification rate. The heterogeneity of emulsions limits the accuracy of dynamic characterization of the emulsification process and the determination method of emulsification rate has always been controversial. The conductivity method we proposed can effectively evaluates the emulsification kinetics. This paper provides theoretical guidance for an in-depth understanding of the mechanism and application of cold recovery technology for heavy oil.

## Introduction

With the rapid development of the petroleum industry in recent years, China’s light crude oil resources have been depleting. As a result, the focus has shifted towards the development and utilization of heavy oil^1^. Successful commercial heavy oil recovery technologies can be divided into thermal and cold recovery technologies^2^. At present, chemical flooding cold oil recovery technology has attracted wide attentions due to its simple operation, low energy consumption and low cost^3–7^. In this process, natural interfacial active ingredients and additional surfactants in heavy oil, will inevitably bring about oil emulsification in water in the formation^8–11^. Thus, the crude oil emulsion formation and stability mechanism, controlling factors and emulsification kinetics are of great significance to the development of heavy oil cold recovery technology^12–17^.

Studies have been extensively conducted to investigate the effect of various factors, such as water cut, temperature, shearing intensity, pH, salt content and chemical additives, on the formation, stability and fluidity of crude oil emulsion^18–24^. Ryoichi et al.^18^ found that the repulsive force increased under low salinity conditions, and the emulsion was more stable under low salinity and high pH. Hongli Chang et al.^19^ studied the effects of water cut, polymer status and concentration, demulsifier type and concentration on the stability of heavy oil emulsion. Pal et al.^20^ investigated nanoemulsion droplet stability by Synergistic Effects of the Gemini Surfactant, PHPA Polymer, and Silica Nanoparticle. Naeeni et al.^21^ discussed the effects of agitation speed, and oil type and volume fraction on mixing characteristics of dilute oil in water dispersions in a stirred tank. Kundu et al.^22^ analysed the effects of temperature and electrolytes on stability of diesel oil-in-water emulsions and its destabilizing mechanisms. The phase behavior of the emulsions under the flow condition was dynamic and the phase transition was observed in complex underground situations^23,24^. Researchers^25–30^ found that the main factors determining the flowability of heavy oil emulsion are temperature, reservoir permeability, water content, and the addition of emulsified viscosity reducer and phase transition of emulsion such as the W/O emulsion to W/O/W in formation pores medium was observed.

Due to the heterogeneity, opacity and thermodynamic instability of emulsion, No well-established dynamic evaluation methods with good reproducibility and accuracy for emulsification process can be obtained. The existing quantitative assessment methods are mainly based on changes of certain emulsion properties such as turbidity^31–33^, particle size^34–36^, conductivity^37–42^, local components of complex microemulsions^43^, rheological property-particle size^44,45^, dispersed oil amounts^46^, reflectance^47^ etc. over time during emulsification. Among these methods, some^31–36^ are not suitable for dark and opaque emulsions, and some^43^ do not work for viscous, multi-component fluids such as real crude oils. The conductivity method is adopted by us because of its simple, nondestructive, cost-effective, easy to implement on-line and widely applicable advantages, further because its reliability to monitor the evolution of the number or concentration of particles in emulsions has been demonstrated^41,42^.

The mechanism of emulsion formation includes several sub-steps, such as droplet rupture and coalescence, the transport of surfactants molecular to the O/W interface, emulsifier’s interfacial diffusion, interfacial adsorption and interfacial arrangement, etc.^44–46,48,49^. The experimental conditions and processing variables impacted on emulsion parameters resulting in dramatic differences in the emulsification mechanisms and kinetics. Baravian et al.^48^ found that emulsion formation was described by dividing into four successive steps: flow start-up, premix formation, progressive reduction in droplet size and changes in droplet size at large enough capillary number and constant shear rate. Sa´nchez et al.^45^ discussed the kinetics of emulsification in terms of two stages: breakup of droplets and transport (and adsorption) of surfactant molecules to the o/w interface. Liu et al.^46^ suggested the formation of crude oil-in-water emulsions under different conditions via two stages: rapid emulsification and emulsion maturation. Beyranvand et al.^50^ found that the rate of emulsion formation was controlled by diffusion induced by the osmosis imbalance condition in LSW injection.

The emulsification rate is usually defined in terms of changes in the torque, conductivity, or particle size over time^46^. Many studies have discussed the kinetics of sub-steps mentioned above. In these studies, kinetics equations (usually, first-order kinetic deduction) have been constructed for the separated droplet rupture and coalescence process^51–56^. The overall emulsification kinetics is more important to the understanding of heavy oil emulsification process in reservoir and the development of heavy oil emulsification recovery technology because overall kinetics promised a full view to emulsification process.

To the best of our knowledge, rare studies focused on the overall emulsification kinetics quantitatively. Mudeme et al.^57^ proposed fitting equation for the overall first-order kinetics of droplet size evolution in highly concentrated emulsions. Pal et al.^20^ found that diameter of oil droplets conforms to an exponential decay function of ultrasonication time based on the modified Hinze theory approach. Alade et al.^13^ presented first-order kinetics and proposed a simple Arrhenius based rate equation to obtain the emulsification kinetic parameters (E_a_ and A_f_) at different temperatures. Liu et al.^46^ put forward a second-order overall kinetics model for emulsification of heavy oil in water.

In that work, we performed emulsification of model oil prepared from mixing heavy oil and its active components with toluene in water within agitated vessels and investigated the effects of various factors such as active components, emulsifier concentration, temperature, pH and stirring speed on emulsification behaviors by online water phase conductance measurement. According to varies of conductance in water phase over time during emulsification, the emulsification rate was characterized and the kinetic equation was derived. Experimental results were discussed qualitatively and quantitatively on the basis of physical phenomena. Such study contributes to emulsifier screening, deeper comprehension of emulsification characteristics, emulsification condition optimization and oil displacement mechanism in chemical flooding cold production.

## Experimental section

### Reagents and instruments

Dodecyl dimethyl betaine (BS-12, AR, Products of Shanghai Maclin Biochemical Technology Co., Ltd), Toluene (AR, Sinopharm Group Chemical Reagent Co., Ltd.), Crude oil was Gudao heavy oil (density 0.9282 g/mL, viscosity 965 mPa s at 50 °C), The experimental water was secondary distilled water. The crude oil underwent separation into functional group components through ion-exchange chromatography, with their compositions and properties shown in Table 1.

**Table 1:** The composition and properties of island heavy oil and its functional group components.

Component	Element content/%					Acid value/mgKOH g^−1^	Relative molecular mass
	C	H	S	N	O		
Heavy oil	82.69	11.60	1.19	0.47	4.05	6.96	524
Acid fraction	82.95	9.36	1.21	0.36	6.12	32.51	749
Alkaline fraction	84.10	11.62	0.49	2.61	1.18	1.28	1012
Amphoteric fraction	79.66	9.14	2.31	1.96	6.93	26.13	1498
Neutral fraction	85.67	13.23	0.21	0.11	0.78	3.21	403

DDSJ-308F conductivity meter (Shanghai Yi Electrical Scientific Instruments Co., Ltd), JJ-1B Electric mixer (Jintan District Xicheng Xinrui instrument factory), Thermostatic magnetic stirrer (Changzhou Danrui experimental equipment Co., Ltd), BA-31 triocular projection polarizing microscope (Macaudy Industrial Group Co., Ltd).

### Experimental methods

#### Separation of functional group components in heavy oil

**Figure 1:**
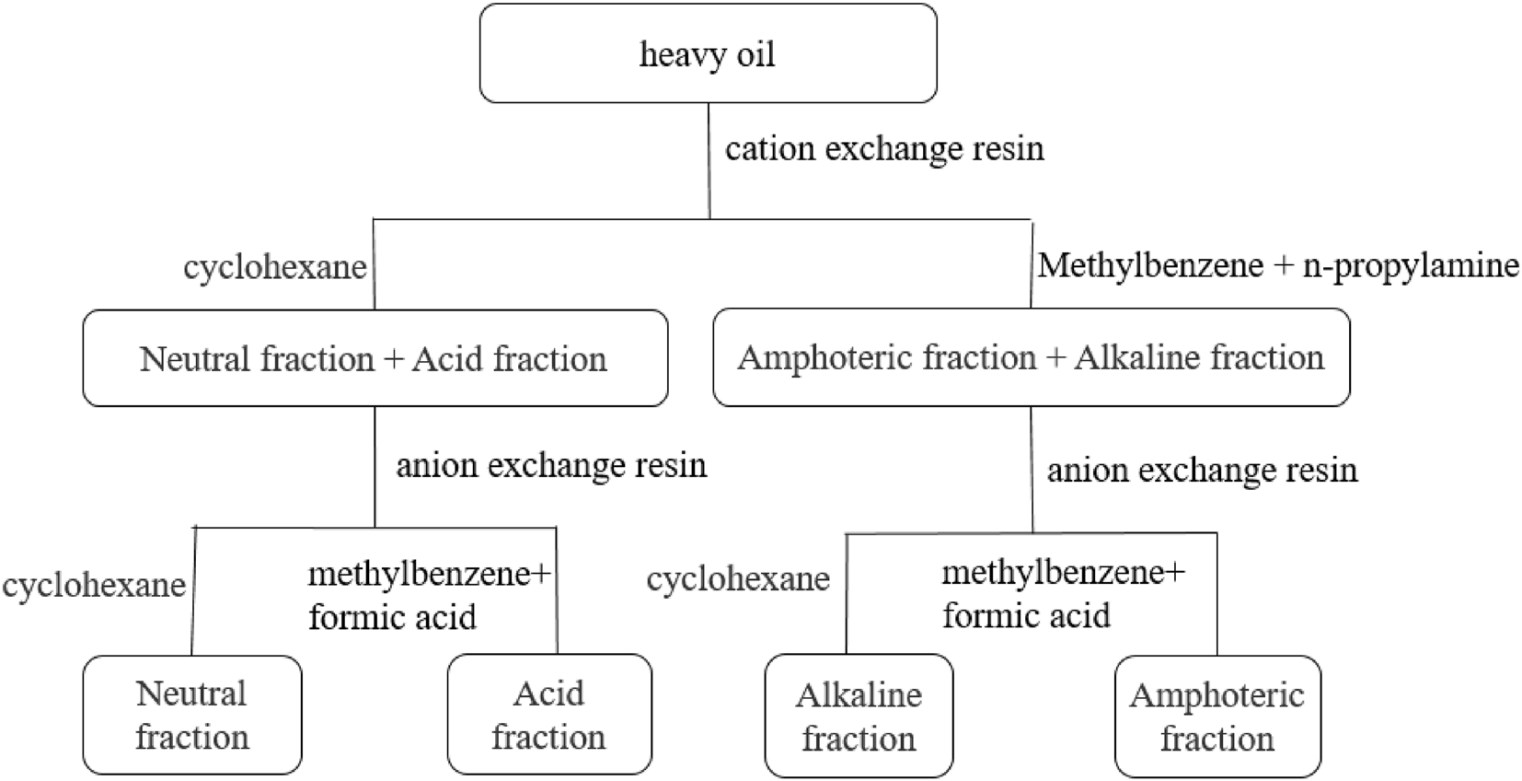
Flow chart of four-component separation of heavy oil functional groups.

The separation method of ion exchange chromatography is based on the functional groups of substances. Heavy oils can be divided into acidic, alkaline, amphoteric and neutral fractions. The process of separation of four components of heavy oil functional groups by ion exchange chromatography^58^ is shown in Fig. 1.

#### Measurement of electrical conductivity

**Figure 2:**
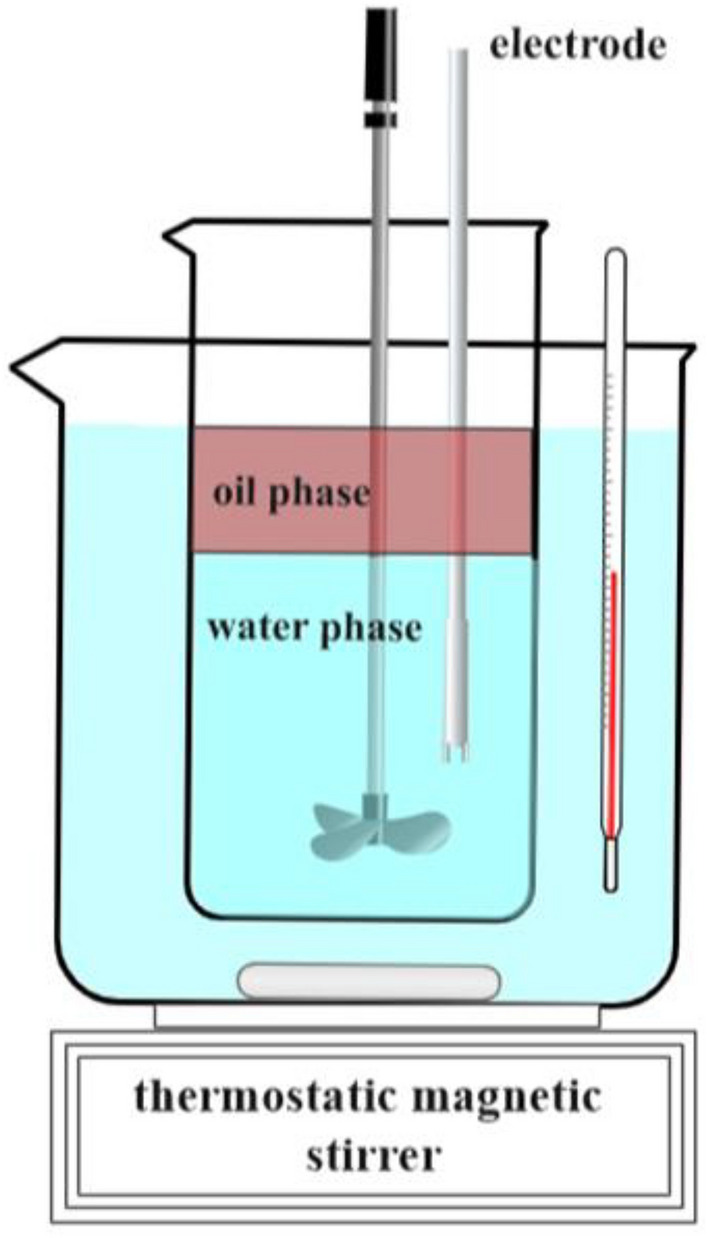
Diagram of the experimental setup for conductivity determination in an emulsification process.

The heavy oil and its functional groups were separately dissolved into a toluene solution with a mass fraction of 0.05%. Dodecyl dimethyl betaine (BS-12) was prepared as a water solution with a concentration of 0.05 mol/L. The experimental device is shown in Fig. 2. The model oil phase and water phase were combined in the internal beaker in a volume ratio of 1:4 (oil to water). After temperature stabilization at 25 and a 30-min incubation period, the electric mixer and conductivity meter were activated, and the oil–water phase began to mix and emulsify to form an oil-in-water emulsion, with conductivity data measured every 20 s.

#### Observation of emulsion morphology

After the conductivity measurement, an appropriate amount of emulsion was taken immediately to prepare a microscope observation slide. The polarizing microscope was then employed to observe the microscopic morphology of the emulsion, and the corresponding images were recorded.

### Establishment of emulsification kinetics equation

**Figure 3:**
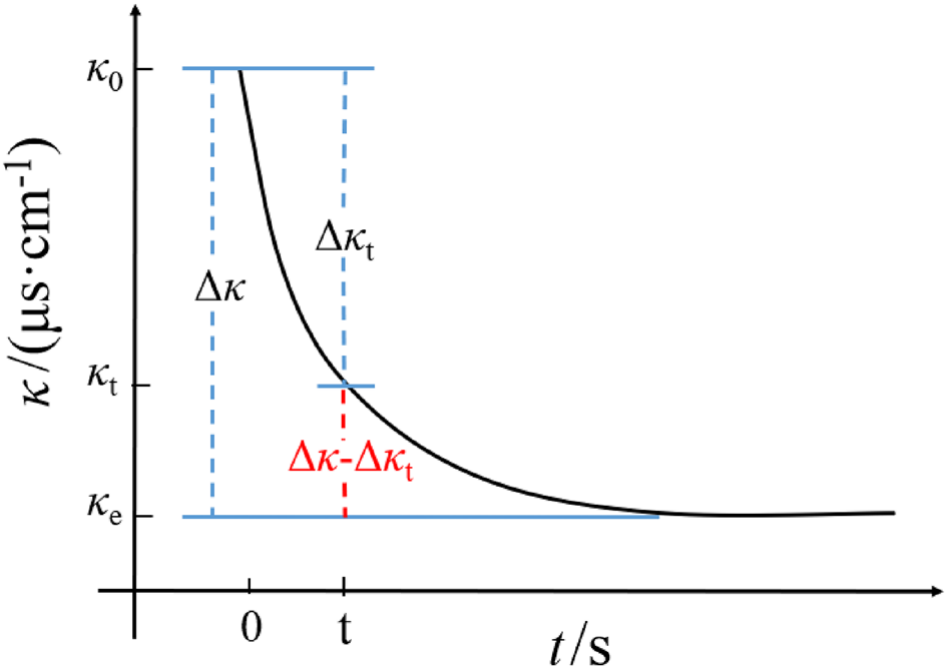
Schematic diagram of the change of aqueous conductivity during emulsification.

Figure 3 illustrates the schematic changes in water phase conductivity during the emulsification process. Due to the mixing of the oil phase, the conductivity of the water phase gradually decreases. Once a uniform and stable emulsion is formed, the conductivity will remain dynamically constant. By substituting the experimental data into the kinetic equations of different reaction order, it is found that the characteristics of second-order reaction kinetics are consistent, and the kinetic equations are established accordingly:

1 $$\begin{array}{*{20}c} {\frac{{\user2{d\Delta \kappa }_{{\varvec{t}}} }}{{{\varvec{dt}}}} = {\text{k}}\left( {\user2{\Delta \kappa } - \user2{\Delta \kappa }_{{\varvec{t}}} } \right)^{2} .} \\ \end{array}$$

When *t* = 0, *Δκ*_*t*_ = 0. the integral of formula (1) is:

2 $$\begin{array}{*{20}c} {\frac{1}{{\Delta \kappa - \Delta \kappa_{t} }} - \frac{1}{\Delta \kappa } = {\text{k}}t.} \\ \end{array}$$

It is deduced that:

3 $$\begin{gathered} \begin{array}{*{20}c} {\frac{t}{{\Delta \kappa_{t} }} = \frac{1}{\Delta \kappa }t + \frac{1}{{{\text{k}}\Delta \kappa^{2} }}} \\ \end{array} \hfill \\ (\Delta \kappa \, = \,\kappa_{0} - \kappa_{{\text{e}}} , \, \Delta \kappa_{{\text{t}}} \, = \,\kappa_{0} - \kappa_{{\text{t}}} ) \hfill \\ \end{gathered}$$

It can be seen from Eq. (3) that the *t/Δκ*_*t*_ of the second-order emulsification reaction has a linear relationship with *t*. According to the change in water conductivity at different time intervals, the relationship curve of *t/Δκ*_*t*_ against t was drawn for linear fitting. The emulsification rate constant *k* was determined according to the slope and intercept.

In the formula:

*Δκ* is the maximum electrical conductivity change, μs cm^−1^;

*Δκ*_*t*_ is the change of conductivity at time t, μs·cm^-1^;

*k* is the emulsification rate constant, μs·(cm·s)^-1^;

*t* is the emulsification time, s;

*κ*_*0*_ is the value of initial conductivity, μs·cm^-1^;

*κ*_*t*_ is the value of conductivity at time t, μs·cm^-1^;

*κ*_*e*_ is the value of electrical conductivity at emulsification equilibrium, μs·cm^−1^.

## Results and discussion

### Effect of heavy oil components on emulsification rate

**Figure 4:**
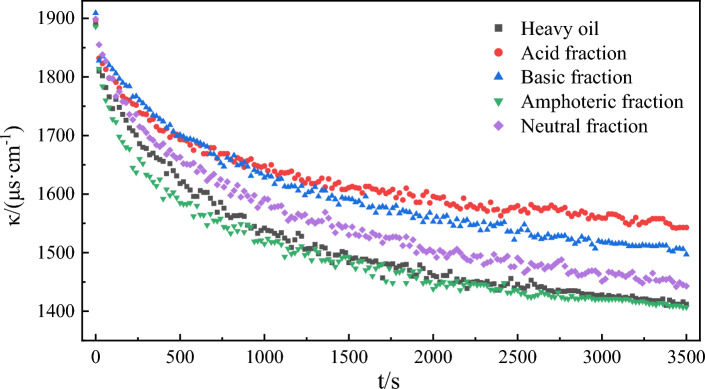
Changes in conductivity over time during emulsification of functional group components of different heavy oils.

**Figure 5:**
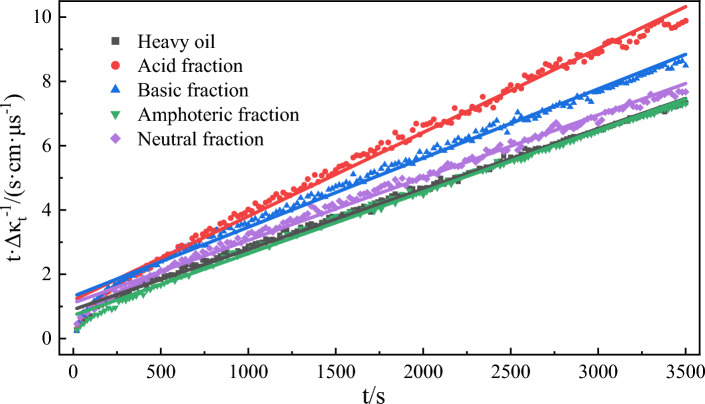
Kinetic fitting curves of emulsification processes of functional components of different heavy oils.

Figure 4 shows the change curve of water phase conductivity over time during emulsification of different active components of heavy oil. With the high-speed mixing of the two phases of oil and water, the emulsion is formed. The electrical conductivity of the crude oil emulsion depends on its water content and the dispersion degree of the water particles^59^. With the emulsification process, the oil phase is dispersed in the water phase, and the conductivity of the water phase decreases. When the emulsion is stable, the water phase conductivity will remain dynamically stable. The rate of conductivity change can quantitatively characterize the emulsification rate. Equation (3) was employed to fit the curves of conductivity changes over time during the emulsification of different model oil and water phases. The fitting results (Fig. 5 and Table 2) showed that the correlation coefficient R^2^ for each fitting curve under the experimental conditions was greater than 0.994. This indicates a strong fit between the experimental results and the assumed emulsification equation. The simulated emulsification process of the oil phase and water phase conforms to the second-order reaction equation. The order of the emulsification rate constant k in the simulated oil-phase emulsification process of different heavy oil components is as follows: acid fraction > amphoteric fraction > heavy oil fraction > alkaline fraction > neutral fraction. Acidic and amphoteric fractions can form a stable interfacial film at the oil–water interface^60^, inhibiting coalescences between droplets. This supports the formation and stability of emulsions to induce a faster, emulsification rate during the stirring process. The kinetic fitting values of *Δκ* for each model oil component are greater than the experimental values, indicating a certain error between the two. Because of the highly conditional reaction emulsification, it is difficult to achieve an ideal emulsification state. The *Δκ* measured in experiments is lower than the fitting value, with a maximum error of 9.31% (for the alkaline fraction), which is considered acceptable.

**Table 2:** Kinetic fitting results of emulsification process of different heavy oil functional group components.

Component	Second-order reaction			*k*/(μs cm^−1^ s^−1^)	Fitting value of Δκ/(μs cm^−1^)	Experimental value of Δ*κ*/(μs cm^−1^)	Error of Δκ/%
	slope	intercept	R^2^				
Model oil	0.0019	0.9416	0.9976	4.01 × 10^–6^	526	477	9.31
Acid fraction	0.0026	1.2540	0.9943	5.65 × 10^–6^	384	354	7.81
Alkaline fraction	0.0021	1.3826	0.9947	3.68 × 10^–6^	454	412	9.25
Amphoteric fraction	0.0019	0.7516	0.9971	5.01 × 10^–6^	526	479	8.93
Neutral fraction	0.0019	1.1491	0.9947	3.61 × 10^–6^	500	456	8.80

**Figure 6:**
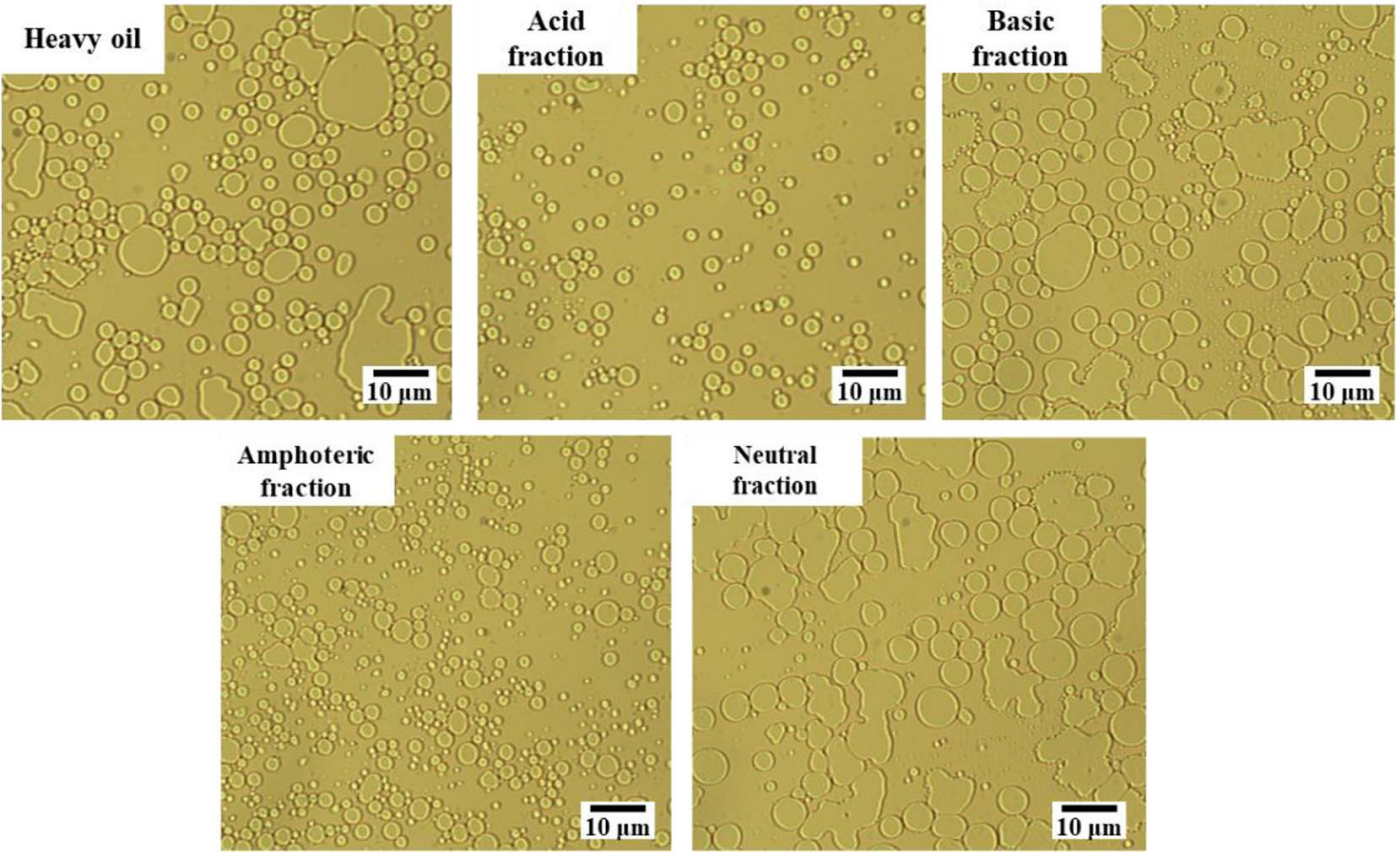
Microscopic image of emulsions of functional group components of different heavy oils.

Since droplet particle size has a significant effect on the agglomeration stratification of emulsion, the particle size of the dispersed phase of emulsion serves as a crucial index for the evaluation of the formation results of an emulsion. The larger the droplet size, the greater the probability of collision and polymerization, which is detrimental to the formation of stable emulsions^61^. Therefore, optimal emulsification conditions should facilitate the generation of small and uniform droplets. It can be seen from the microscopic images of emulsions with different functional groups of heavy oil in Fig. 6 that emulsions formed by acidic and amphoteric fractions exhibit smaller and more uniform droplet size, coupled with faster emulsification rates. Conversely**,** emulsions formed by the neutral fraction display large, uneven droplets with a tendency towards fusion and aggregation. This results in a less stable emulsion and unfavorable emulsification results.

### Effect of emulsifier concentration on emulsification rate

**Figure 7:**
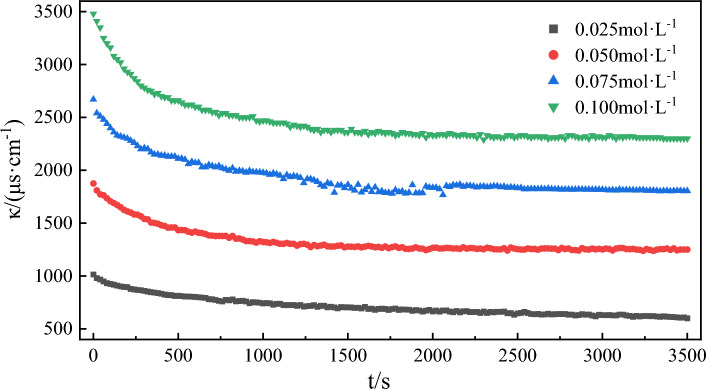
The change of conductivity over time during model oil emulsification was simulated at different emulsifier concentrations.

**Figure 8:**
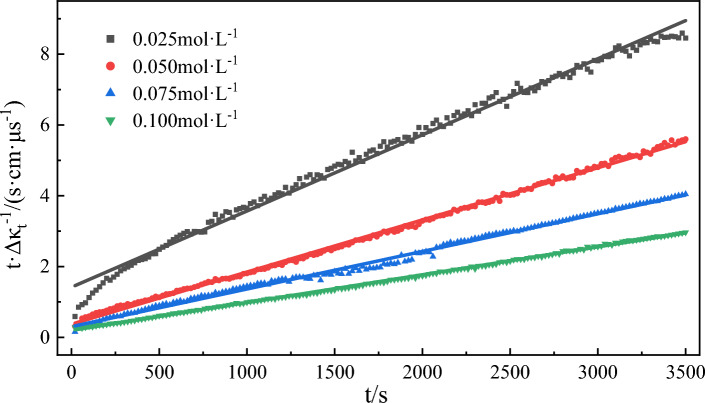
Kinetic fitting curves of emulsification processes with different emulsifier concentrations.

Equation (3) was employed to fit the water phase conductivity of emulsifiers at different concentrations. The results are shown in Table 3 and Fig. 7. The analysis indicates that the correlation coefficients of the fitting curves are greater than 0.991, affirming the suitability of this fitting equation for the data (Fig. 8). With an increase in emulsifier concentration, the emulsifying rate constant the emulsifying rate constant first rose and reached its maximum value at a concentration of 0.05 mol/L before decreasing. When the emulsifier concentration is below 0.05 mol/L, its increase causes higher adsorption of emulsifier molecules at the oil–water interface, facilitating the mixing of oil and water phases and the emulsification process. However, at a concentration of 0.05 mol/L, the emulsification rate constant reaches a maximum due to the saturation in interfacial facial mask adsorption^62,63^. Further increase in the emulsifier concentration after 0.05 mol/L results in a decrease in the emulsification rate constant and a slowdown in the emulsification rate.

**Table 3:** Kinetic fitting results of emulsification process with different emulsifier concentrations.

Emulsifier concentration/(mol L^−1^)	The second order reaction			*k*/(μs cm^−1^ s–^−1^)	Fitting value of *Δκ*/(μs cm^−1^)	Experimental value of *Δκ*/(μs cm^−1^)	Error of *Δκ*/%
	Slope	Intercept	R^2^				
0.025	0.0022	1.4168	0.9919	3.41 × 10^–6^	454	414	8.81
0.050	0.0015	0.3727	0.9981	6.03 × 10^–6^	666	625	6.56
0.075	0.0011	0.3021	0.9955	4.00 × 10^–6^	909	865	4.84
0.100	0.0008	0.0008	0.9991	3.27 × 10^–6^	1250	1180	5.60

**Figure 9:**
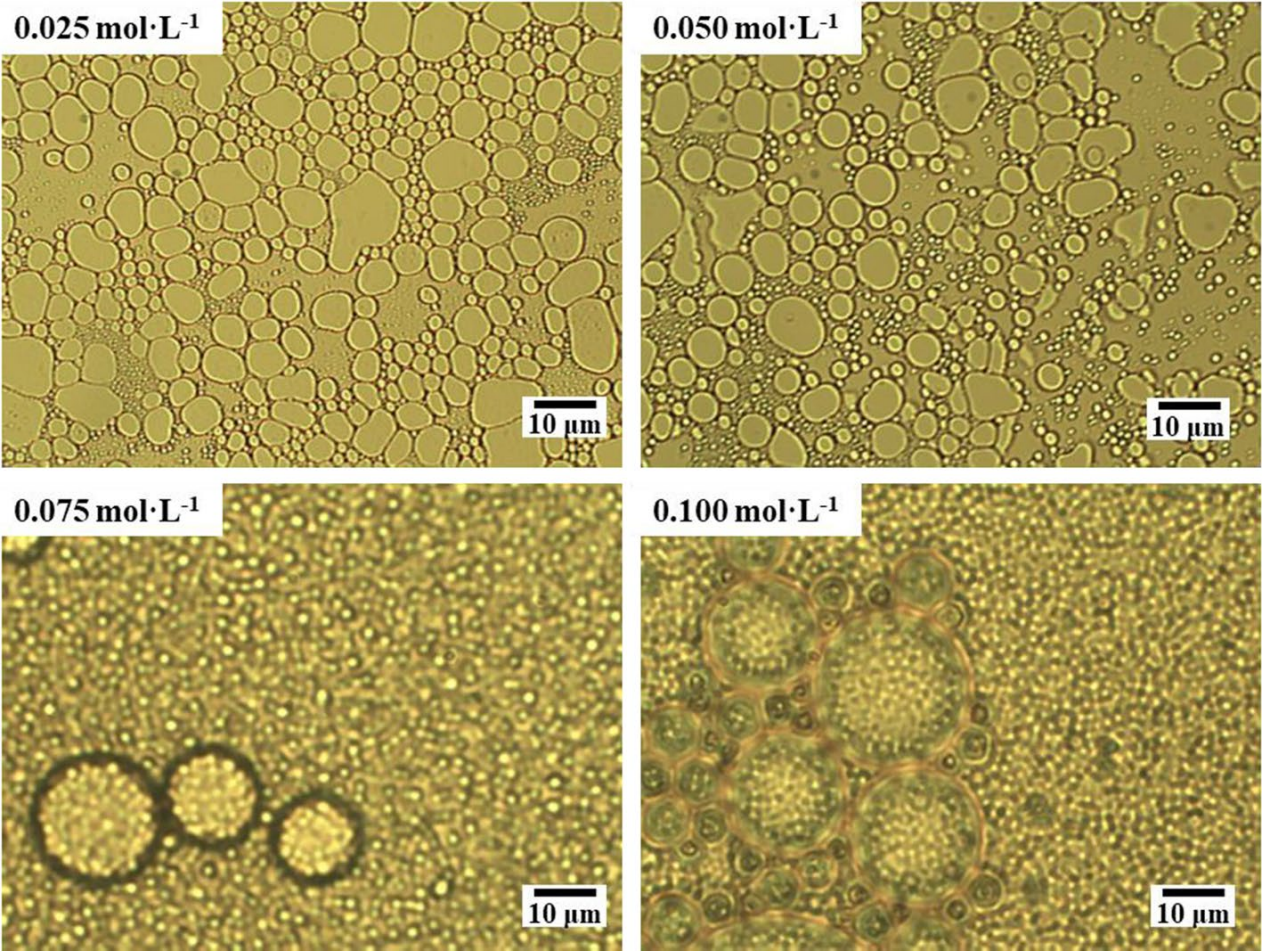
Microscopic image of model oil emulsion at different emulsifier concentrations.

From the microscopic images of model oil emulsions under different emulsifier concentrations in Fig. 9, it can be seen that as the emulsifier concentration increases, the particle size of the emulsion droplets decreases, while the number of droplets increases. However, there is a point beyond which the particle size is difficult to further reduce. When the emulsifier concentration increases after 0.05 mol/L, it tends to flocculate into vesicles or micelles^64^. This will increase the solubility of excess emulsifier molecules, which is not conducive to their adsorption at the interface, hinder the emulsification process, and reduce the rate constant.

### Effect of temperature on emulsification rate

**Figure 10:**
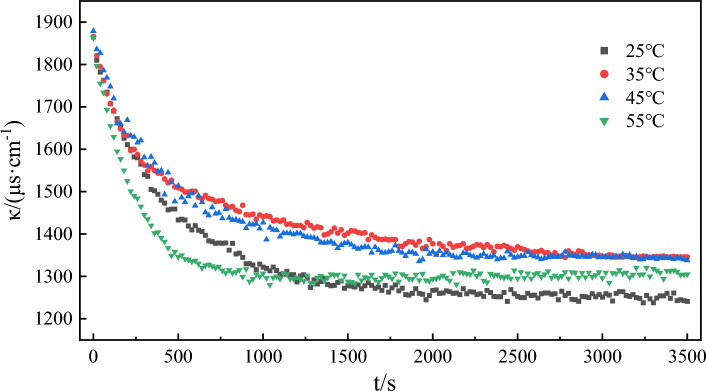
The change of conductivity over time during model oil emulsification at different temperatures.

**Figure 11:**
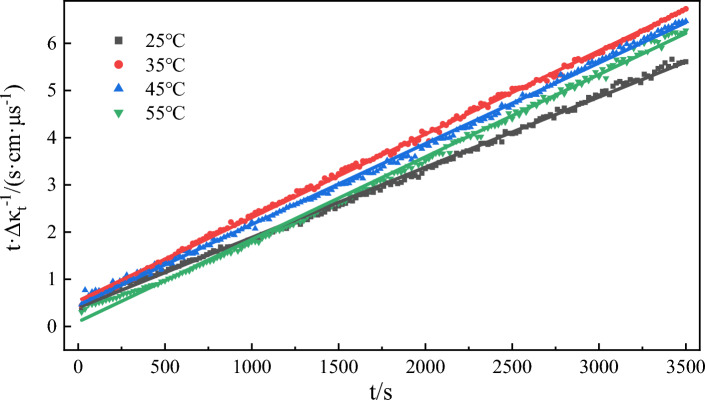
Kinetic fitting curves of emulsification processes at different temperatures.

The change curve of water phase conductivity with time in the emulsification process of model oil at different temperatures is shown in Fig. 10. The data of this group are fitted to Eq. (3), and the fitting results are shown in Fig. 11 and Table 4. All the fitting curves exhibited correlation coefficients greater than 0.997 at different temperatures. This suggests that the emulsification process conforms to the assumed Eq. (3) and follows a second-order rate reaction. As can be seen from Table 4, with the increase in temperature, the emulsification rate constant also rises. Notably, this increase is more greater at higher temperatures. The colloidal stable system with asphaltene as the core in model oil is no longer stable at high temperature and shows signs of dispersion^65^. The higher the temperature is, the more dispersed the micelles will eventually disintegrate, resulting in lower viscosity of crude oil, increased rheology, and easier mixing with water. Under high temperatures, the solubility of the active components (acidic and amphoteric fractions) in petroleum increases^66^, which will change the strength and toughness of the oil–water interface film and accelerate the emulsification process. According to the Arrhenius formula, $lnk = - \frac{{E_{a} }}{RT} + lnA$, the activation energy *E*_*a*_ = 40.282 kJ/mol for the model oil emulsification reaction can be obtained by through one-dimensional linear regression method.

**Table 4:** Kinetic fitting results of emulsification processes at different temperatures.

Temperature/℃	The second order reaction			*k*/(μs cm^−1^ s^−1^)	Fitting value of *Δκ*/(μs cm^−1^)	Experimental value of *Δκ*/(μs cm^−1^)	Error of *Δκ*/%
	Slope	Intercept	R^2^				
25	0.0015	0.3960	0.9980	5.68 × 10^–6^	666	624	6.31
35	0.0018	0.5409	0.9991	5.99 × 10^–6^	555	520	6.31
45	0.0017	0.4496	0.9984	6.42 × 10^–6^	588	541	7.99
55	0.0017	0.0966	0.9975	2.96 × 10^–5^	588	558	5.10

**Figure 12:**
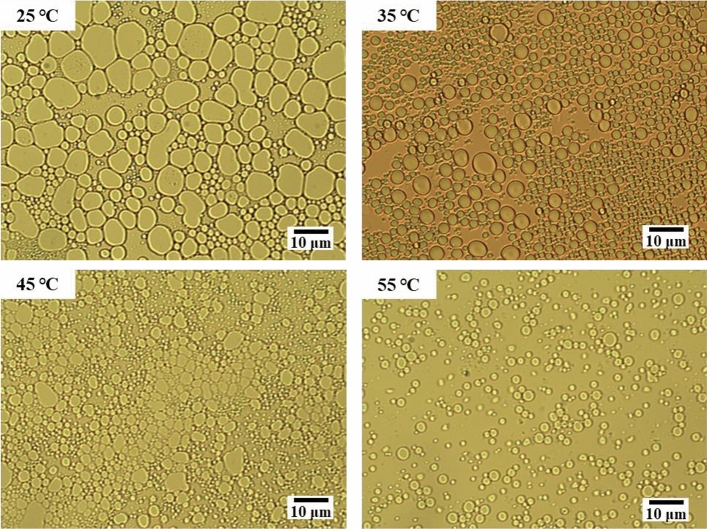
Microscopic image of model oil emulsion at different temperatures.

It can be seen from the microscopic images of model oil emulsions at different temperatures in Fig. 12 that as the emulsification temperature increases, the droplets of model oil emulsions become smaller, exhibiting more uniform particle size, and a narrower particle size distribution. Higher temperature proves beneficial to the emulsion formation, with the emulsification rate increasing alongside. When emulsified for the same time, the smaller the average particle size of the formed emulsion, the greater the propensity for a uniform and stable model oil emulsion to form^67^.

### Effect of pH of water phase on emulsification rate

**Figure 13:**
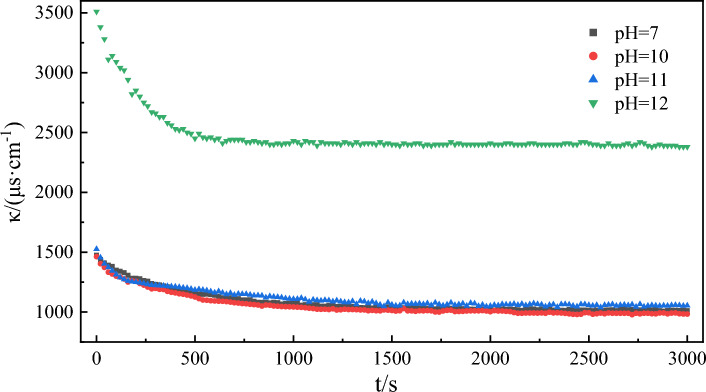
Simulation of conductivity over time during model oil emulsification at different pH.

**Figure 14:**
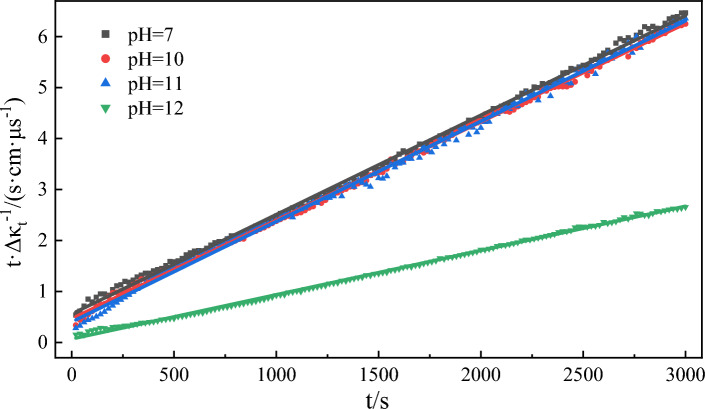
Emulsion process kinetic fitting curves at different pH.

Alkali flooding is a common method to enhance tertiary oil recovery. The production of heavy oil is increased by in-situ emulsification of surfactant generated by the reaction of injected alkaline solution with natural organic acid in heavy oil^64^. Therefore, the alkalinity of the water phase bears a significant correlation with the formation of heavy oil emulsions. The curve (Fig. 13) representing the conductivity of the water phase over time under different pH conditions was fitted to Eq. (3). The results (Fig. 14 and Table 5) showed that the correlation coefficients of the experimental fitting curves were all greater than 0.997. This indicates that the fitting results are highly satisfactory. The emulsification rate constant exhibited an increase with the rise in the pH in the water phase. Given that heavy oil contains many acidic functional groups^28^, they serve as the key factors in emulsion formation. When the pH is greater than 7, the acidic groups in the heavy oil can be activated by alkali, leading to a saponification reaction for the formation of a surfactant that exerts a certain emulsification effect^68^, thereby accelerating emulsion formation.

**Table 5:** Kinetic fitting results of emulsification process at different pH.

pH	The second order reaction			*k*/(μs cm^−1^ s^−1^)	Fitting value of *Δκ*/(μs cm^−1^)	Experimental value of *Δκ*/(μs cm^−1^)	Error of *Δκ*/%
	Slope	Intercept	R^2^				
7	0.0020	0.5591	0.9984	7.15 × 10^–6^	500	464	7.20
10	0.0019	0.4659	0.9988	7.75 × 10^–6^	526	480	8.74
11	0.0020	0.4026	0.9975	9.94 × 10^–6^	500	472	5.60
12	0.0009	0.0692	0.9986	1.17 × 10^–5^	1111	1130	1.71

**Figure 15:**
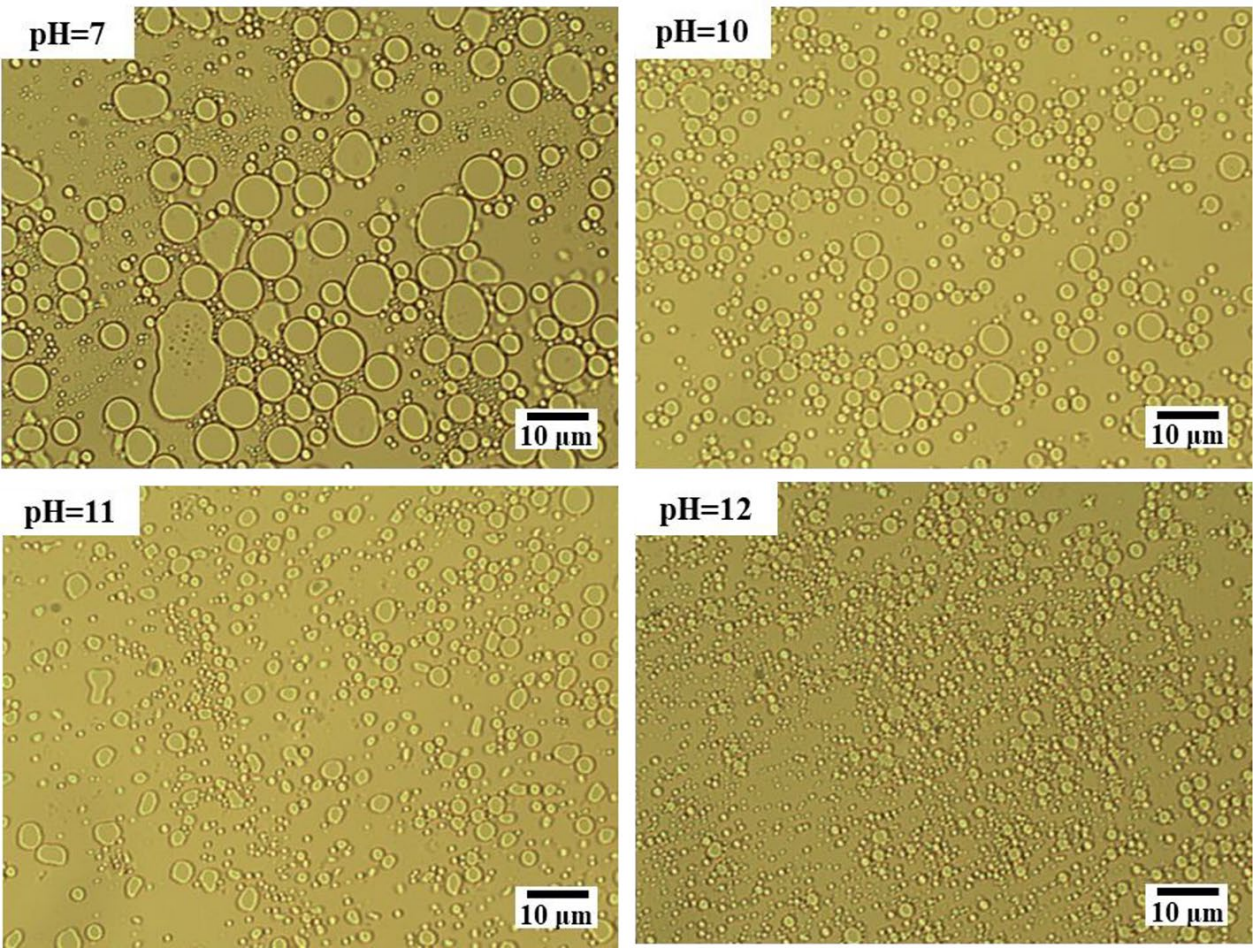
Microscopic images of model oil emulsions at different pH.

From the microscopic images of model oil emulsions formed under different pH conditions in Fig. 15, it can be seen that the average particle size of the emulsion formed at pH = 7 is much larger than that at pH = 10, 11, and 12. At pH = 7, the emulsion mainly depends on the energy of stirring. The effect of surfactant is minimal, with a slower emulsification rate and larger particle size of the emulsion formed within the same duration. At pH levels of 10, 11, and 12, the surfactant formed by the reaction of the acidic component of the model oil with the alkali exhibits a favorable emulsification effect^69^. This substance synergizes with the external stirring to accelerate emulsification and yield smaller emulsion droplets. It becomes clear that the greater the pH of the water phase, the better the emulsification effect.

### Effect of stirring speed on emulsification rate

**Figure 16:**
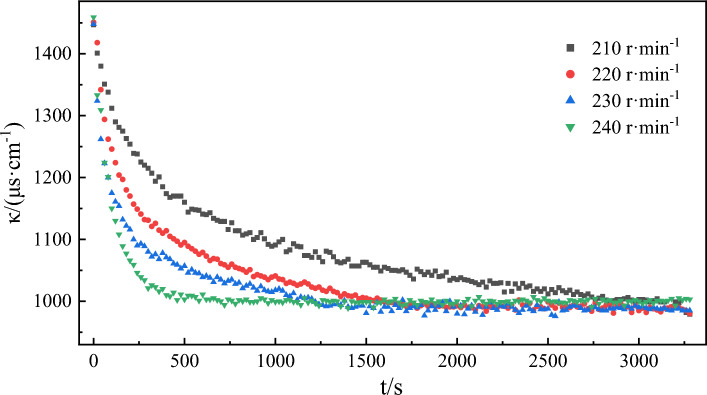
The change of conductivity over time during the emulsification process of model oil is simulated at different speeds.

**Figure 17:**
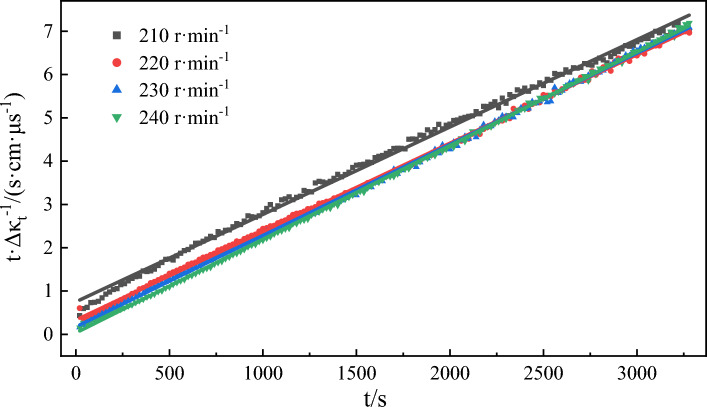
Kinetic fitting curves of emulsification processes at different speeds.

Figure 16 shows the variation curve of conductivity over time during emulsification at different stirring speeds. In general, the emulsification process does not occur spontaneously. The mixing and stirring of the oil phase and water phase (i.e., providing mixing energy) is one of the necessary conditions for emulsion formation^70^. In the stirring process, the oil phase undergoes deformation and elongation and breaks into smaller oil droplets that ultimately coalesce to form an emulsion^71^. The change curves of emulsion conductivity over time at different stirring speeds were fitted to Eq. (3). The fitting results (Fig. 17, Table 6) indicate that the correlation coefficients of each fitting curve were greater than 0.997, demonstrating that the experimental data of each group were well-fitted to Eq. (3). When the stirring speed increased from 210 r/min to 240 r/min, the emulsification rate constant also saw a significant rise from 5.32 × 10^–6^ μs/(cm s) to 1.34 × 10^–4^ μs/(cm s), revealing the strong influence of different stirring speeds on the emulsification rate constant. In this experiment, stirring serves as the only external energy for mixing the oil phase and water phase. Different stirring speeds provide different mixing energies, which determines the rate of the emulsification process. The high-speed stirring causes the oil droplets to deform and stretch, and then shear and break into small particles. The increase of stirring speed and time will make the oil droplets of emulsion smaller. And the smaller the particle size of the oil droplets is, the easier it is to get a evenly mixed oil-in-water emulsion. The relative error between the fitted *Δκ* value and the experimental value is the largest when the stirring speed is 210 r/min. At this time, the mixing energy provided by stirring is at its lowest. As a result, the relative emulsification effect is the worst, along with the smallest emulsification rate constant.

**Table 6:** Kinetic fitting results of emulsification process at different speeds.

Stirring speed/(r min^−1^)	The second order reaction			*k*/(μs cm^−1^ s^−1^)	Fitting value of *Δκ*/(μs cm^−1^)	Experimental value of *Δκ*/(μs cm^−1^)	Error of *Δκ*/%
	Slope	Intercept	R^2^				
210	0.0020	0.7524	0.9970	5.32 × 10^–6^	500	468	6.40
220	0.0020	0.3313	0.9992	1.21 × 10^–5^	500	471	5.80
230	0.0021	0.1756	0.9994	2.51 × 10^–5^	476	463	2.73
240	0.0022	0.0341	0.9996	1.34 × 10^–4^	454	456	-0.44

**Figure 18:**
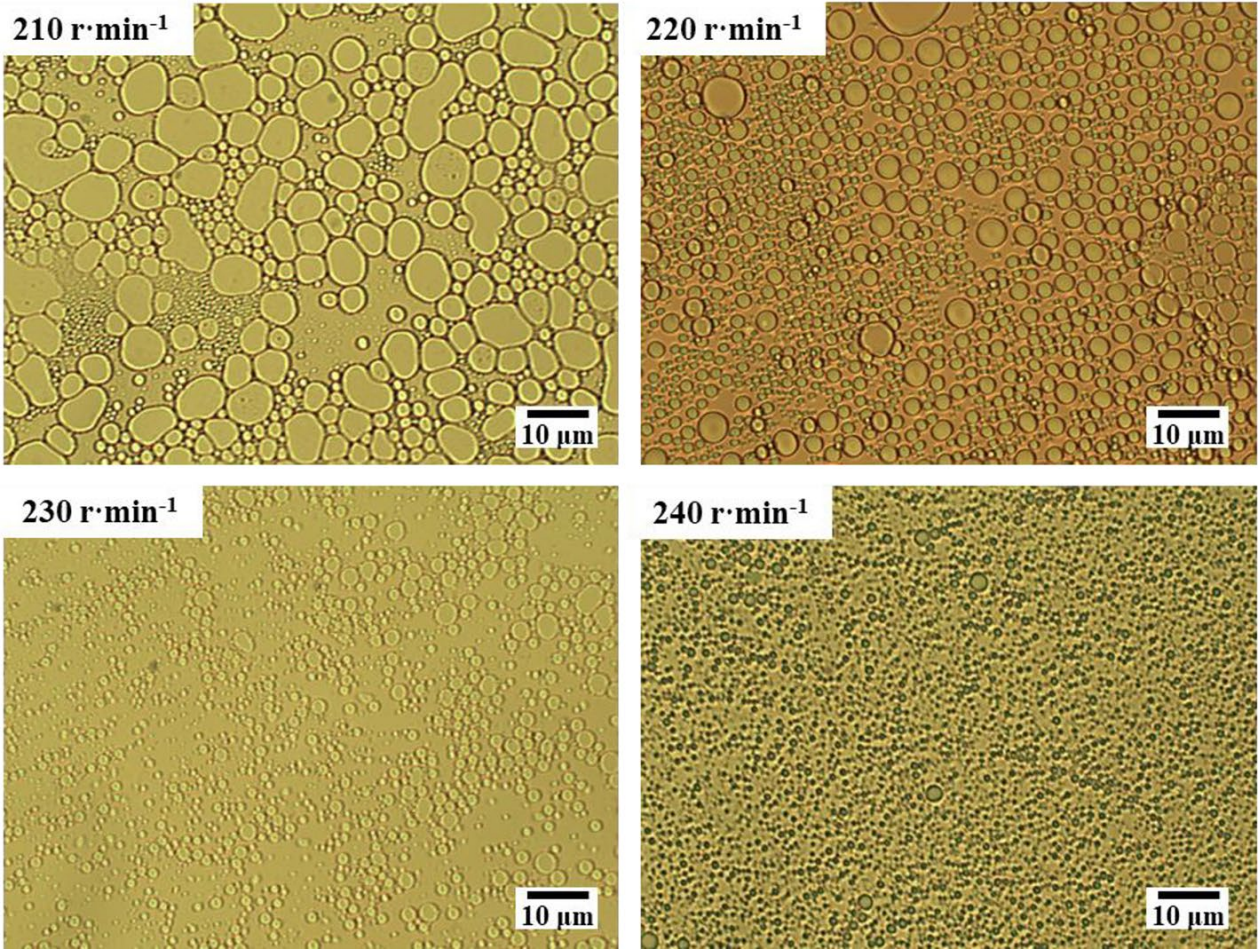
Microscopic image of model oil emulsion at different rotational speeds.

The microscopic images of model oil emulsion at different rotational speeds in Fig. 18 provide further confirmation that the stirring speed is the most important factor affecting the emulsification effect. As the stirring speed increases from 210 to 240 r/min, there is a significant variation in emulsification results. At a stirring speed of 210 r/min, the emulsion exhibits a large average particle size and the droplets have irregular shapes, showing a tendency to coalesce with each other. In contrast, at 240 r/min, the obtained emulsion features a smaller average particle size, uniform droplet sizes, and regular shapes. The emulsification result at high speed is superior at the same time.

## Conclusion

(1)(1) The kinetic model of heavy oil emulsification process was established for the first time by using the conductivity method. It was proposed that the change of water phase conductivity during emulsification conforms to the second-order rate equation:
  $$\frac{{d\Delta \kappa_{t} }}{dt} = {\text{k}}\left( {\Delta \kappa - \Delta \kappa_{t} } \right)^{2}$$
  The emulsification data under different experimental conditions were well-fitted, with linear correlation coefficients all greater than 0.991, indicating that the change in water phase conductivity in the emulsification process conformed to the second-order reaction equation. The conductivity method has the advantages of simple, non-destructive, low cost, easy on-line implementation and wide application, which provides theoretical guidance for understanding the mechanism and application of heavy oil cold production technology.
(2)(2) Acidic and amphoteric fractions can form a stable interfacial film at the oil–water interface, this prevents coalescence between droplets, and facilitates the formation and stability of the emulsion, resulting in a faster emulsification rate during the stirring process. With an increase in emulsifier concentration, the emulsifying rate constant the emulsifying rate constant first rose and reached its maximum value at a concentration of 0.05 mol/L before decreasing. The emulsification rate constant continues to increase with rising temperatures. The higher the temperature, the greater the increase in emulsification rate constant. The activation energy for the heavy oil emulsification reaction is calculated to be *E*_*a*_ = 40.282 kJ/mol. In the emulsification process of the oil phase and water phase, the stronger the alkalinity of the water phase, the larger the emulsification rate constant and the emulsification rate. Different stirring speed strongly affects the emulsification rate constant, with the faster stirring speed leading to larger emulsification rate.

## Data Availability

Data will be provided on request from the corresponding author.
